# Genetic Relationships Among Resilience, Fertility and Milk Production Traits in Crossbred Dairy Cows Performing in Sub‐Saharan Africa

**DOI:** 10.1111/jbg.12933

**Published:** 2025-03-03

**Authors:** R. D. Oloo, R. Mrode, C. C. Ekine‐Dzivenu, J. M. K. Ojango, J. Bennewitz, G. Gebreyohanes, A. M. Okeyo, M. G. G. Chagunda

**Affiliations:** ^1^ Animal Breeding and Husbandry in the Tropics and Subtropics University of Hohenheim Stuttgart Germany; ^2^ Livestock Genetics International Livestock Research Institute Nairobi Kenya; ^3^ Animal and Veterinary Science Scotland Rural College Edinburgh UK; ^4^ Institute of Animal Science University of Hohenheim Stuttgart Germany; ^5^ Centre for Tropical Livestock Genetics and Health The University of Edinburgh Midlothian UK

**Keywords:** climate change, heat stress, milk yield, robustness, sub‐Saharan Africa

## Abstract

Change in climate over the past years and its impact on the environment have necessitated the inclusion of resilience traits in the breeding objectives of dairy cattle. However, the relationship between resilience and other traits of economic importance in dairy production is currently not well known. This study examined the genetic parameters and relationships among resilience, fertility and milk production traits in dairy cattle in Kenya. Indicators of general resilience and heat tolerance were defined from the first parity test‐day milk yield records. Indicators of general resilience included variance of actual deviations (*LnVar1*), variance of standardised deviations (*LnVar2*), lag‐1 autocorrelation (*r*_auto_) and skewness (*Skew*) of standardised deviations in milk yield. Heat tolerance indicators at temperature‐humidity index 80 included the slope of the reaction norm (*Slope*), absolute slope of the reaction norm (*Absolute*), and the intercept of the reaction norm model (*Intercept*). Cows with > 50% taurine genes had lower age at first calving (AFC), longer calving intervals (CI) and higher test‐day milk yield (MY). The heritability estimates of AFC, CI and MY were 0.17 ± 0.033, 0.06 ± 0.012 and 0.35 ± 0.021, respectively. The repeatability estimates of CI and MY were 0.06 ± 0.012 and 0.47 ± 0.009, respectively. The low heritability and non‐significant permanent environmental variance of CI showed that CI is heavily influenced by external factors, such as management practices. AFC was negatively genetically correlated with both CI (−0.88 ± 0.077) and MY (−0.53 ± 0.059) showing that animals that attain sexual maturity earlier exhibit longer CI and higher milk production. A positive genetic correlation (0.62 ± 0.077) between CI and MY shows that high‐yielding cows face challenges in maintaining shorter calving intervals. Heritability estimates of nearly all resilience indicators were significant and ranged from 0.05 to 0.34. Heat tolerance indicators showed low to non‐significant genetic correlations with general resilience indicators, suggesting that different genetic factors are involved in responses to different types of disturbances. There was a generally positive genetic correlation between resilience and fertility, implying that resilient animals might have better fertility. All indicators, except *LnVar1* and *LnVar2*, revealed an antagonistic genetic relationship between resilience and milk production. The findings present an opportunity for including resilience in the development and application of selection indices in dairy cattle, especially for the tropics.

## 1 Introduction

Despite the relevance of dairy production in the fight against food insecurity, negative effects of climate change and general changes in the production environment pose enormous challenges to its profitability. These changes are naturally occurring, and their harshness cannot be sustainably addressed through husbandry interventions. Thus, improving the resilience capacity and robustness of dairy cattle to adapt to the changing environments is becoming increasingly important. Resilience is defined as the capacity of animals to be minimally affected by disturbances in their environment or to rapidly recover from the negative effects of exposure to these disturbances (Colditz and Hine 2016). Resilience is closely related to robustness, which is the ability of an animal to express its production potential in a wide range of environmental conditions without compromising its reproduction, health and well‐being (Knap 2005). Recently, this area of research has attracted attention, and indirect phenotypes related to resilience to environmental disturbances in livestock species have been defined (Berghof et al. 2019).

Environmental disturbances affecting livestock species can be grouped into two groups: macro‐environmental and micro‐environmental (Mulder et al. 2013; Falconer and Mackay 1996). Macro‐environmental disturbances are identifiable and can be quantified or categorised (e.g., ambient temperature). Micro‐environmental disturbances occur within a macro‐environment and are either unknown or non‐specific (e.g., social interaction). Defined resilience phenotypes related to microenvironmental disturbances include variance, lag‐1 autocorrelation (*r_auto_*) and skewness (*Skew*) of deviations in production levels (Poppe et al. 2020; Berghof et al. 2019). Variance of deviations captures the severity and duration of environmental disturbances an individual experiences. The *r*_auto_ gives the rate of recovery of an individual animal from disturbances. *Skew* captures the severity of disturbances experienced by an animal. Resilient animals have lower variance of deviations, lower lag‐1 autocorrelation and higher skewness of deviations.

Phenotypes that measure resilience to macro‐environmental disturbances are derived from reaction norm functions, where a disturbance is modelled as a continuous environmental variable. Under this category, the distinct phenotypes include the slope of the reaction norm, which indicates the direction of production in the face of a challenging state of affairs and measures environmental sensitivity (Streit et al. 2013) and the absolute slope of the reaction norm, which indicates stability in production (Sánchez‐Molano et al. 2019). The reaction norm indicators also measure the level of robustness since they quantify the production level of animals at different levels of a disturbance (Streit et al. 2013). A more resilient animal is expected to have a less negative slope and a lower absolute slope of the reaction norm and vice versa (Oloo et al. 2024; Sánchez‐Molano et al. 2019). In addition to the slope functions, the intercept of the reaction norm model, which estimates the general mean production level, might provide additional valuable information about resilience to macroenvironmental disturbances. This is because, whereas a stable production profile amidst environmental disturbance is desirable, animals attaining stability at a higher production level are much more desirable than those that are stable but at a lower level.

Oloo, Mrode, et al. (2023) and Oloo et al. (2024) defined these indicators from first parity milk production and demonstrated that they could effectively quantify the level of general resilience and heat tolerance of dairy cattle performing in sub‐Saharan Africa (SSA). However, dairy productivity is a function of many variables, and resilience is just one of them. Other traits, such as milk quality and quantity, fertility, feed efficiency and growth performance, also affect the net productivity of the dairy enterprise. As such, the overall goal of dairy production should be to produce robust and resilient cattle with the ability to express their production potential in a wider range of environments without compromising their reproduction, growth, health and general well‐being. Ensuring sustainable profitability calls for the right balance among most, if not all, key traits in dairy production. Genetic evaluations provide a pathway to establish this balance and guide multi‐trait selection that allows for simultaneous improvement of more than one trait at the same time.

Therefore, if resilience is to be included in the selection index of dairy cattle, the genetic relationships between resilience phenotypes and other important traits need to be examined. To the best of our knowledge, this has yet to be established in dairy cattle performing in SSA. Building on the work of Oloo, Mrode, et al. (2023); Oloo et al. (2024), this study explored the existing relationships among resilience, fertility and milk production traits in dairy cattle performing in SSA. Thus, the objectives of the study were (i) to estimate genetic parameters of resilience indicators derived from first parity milk yield, age at first calving (AFC), calving interval and milk yield, (ii) to estimate genetic correlations among resilience indicators and (iii) to establish genetic correlations among resilience, fertility and milk production in crossbred dairy cows performing in different agroecological zones of Kenya.

## 2 Materials and Methods

### 2.1 Performance Data

Cow performance data from three different herds, situated in three different agroecological zones of Kenya, hence representing different levels of exposure to natural environmental stresses, were analysed. Two of the herds were performing in the agroecological zone IV (semi‐arid) but in regions of the country where different agricultural practices were predominant. The agricultural practices adopted in the region were thus used to classify the farms as semi‐arid arable (SAA) and semi‐arid pasture‐based (SAP). The third farm was in agroecological zone V (semi‐humid (SH)). The climatic conditions of these agroecological zones and breeding practices adopted in each farm were described by Oloo, Mrode, et al. (2023). Briefly, the SAA zone receives an annual rainfall of 800–950 mm. It has bimodal rains from March to April and November to December. The SAP zone, with an annual rainfall of 650–750 mm, also experiences bimodal rains from March to June and October to December. The SH zone has rainfall ranging from 850 to 1100 mm, has two rainy seasons, with long rain occurring between April and June and short rains between October and December.

In SAA and SAP farms, the crossing of indicine cows with taurine bulls and vice versa is practiced. The subsequent generations are mated with either pure indicine, crossbred or taurine bulls, resulting in a cattle population with varying proportions of different breeds. The main taurine cattle breed used in both farms is Holstein‐Friesian, but Jersey and Ayrshire are also occasionally used. The Zebu breeds used in SAA and SAP are Boran and Sahiwal, respectively. The SH herd consists of composite cattle with 25%–50% Gir genes. The remaining 50%–75% breed proportion is distributed among taurine and indicine breeds, with each breed contributing < 25%. The breeds used to produce these synthetic crossbreds include Gir, Holstein‐Friesian, Ayrshire, Jersey, Guernsey, Brown Swiss, Fleckvieh, Milking Shorthorn, Meuse Rhine Issel and Montbéliarde, Sahiwal and Boran.

The data consisted of 13,203 calving events and 311,356 milk production records of 4310 dairy cows born between 1980 and 2019. From these, three traits were analysed: AFC, calving interval (CI) and test‐day milk yield (MY). The data were subjected to several edits, including the exclusion of cows with AFC below 21 months and above 48 months. The lower limit was based on the possibility of including abortions that occurred in late pregnancy. The upper limit took care of the likelihood of a subsequent calving event being misclassified as the first calving due to unrecorded first calving. Calving interval was restricted to between 300 and 730 days. Milk yield values below 0.5 and above 45 kg per day, as well as days in milk beyond 400, were excluded. The standard limit of 305 days in milk does not reflect the real production patterns of the population studied because, in the study areas, farmers tend to milk their cows longer, upon consecutive failure of cows to conceive in time because of malnutrition‐triggered infertility or poor estrus detection or both (Mrode et al. 2021). Records on the first five parities were considered in these analyses. Age at first calving, calving interval and milk yield values that deviated by more than four standard deviations (SD) from the mean were removed. To correct for year‐season (YS) effects of either birth, calving or milking, contemporary groupings of YS were defined. Each agroecological zone had four seasons that were based on the precipitation pattern (Oloo, Mrode, et al. 2023). Long and short rain periods were considered wet seasons 1 and 2, respectively. A dry period before the long rain was considered dry season 1 and that before the short rains was considered dry season 2. Contemporary groups having less than three records were excluded from the analysis. The final dataset had 3505 AFC, 6191 calving interval and 276,781 test‐day milk yield records from 3505 dairy cows.

These cows were grouped into three breed groups based on the proportion of *Bos taurus* genes in their breed composition using the breed information provided by the farmers: < 50% *Bos taurus* (*n* = 1118), > 50%–87.5% *B. taurus* (*n* = 817) and > 87.5% *B. taurus* (*n* = 1570). The pedigree used in the analysis consisted of 6291 individuals spanning 21 generations, including 728 sires and 3169 dams.

### 2.2 Resilience Indicators

Seven resilience indicators were defined from first parity test‐day milk yield records: log‐transformed variance of actual deviations corrected for average expected milk yield (*LnVar1*), log‐transformed variance of standardised deviations in milk yield (*LnVar2*), lag‐1 autocorrelation of standardised deviations in milk yield (*r*_auto_), skewness of standardised deviations in milk yield (*Skew*), milk production change per unit change in temperature‐humidity index (THI) at THI 80 (*Slope*) and its absolute value (*Absolute*) and the intercept of an individual's response of milk yield to heat load (*Intercept*).

Resilience indicators reflecting changes in milk yield in response to varying heat loads (*Slope*, *Absolute* and *Intercept*) were derived from a random regression model, including a reaction norm shown below.

(1) $$Y=\mathrm{X\beta}+f\left(\rho ,j\right)+f_{i}\left(\alpha ,j\right)+\text{Zysm}+e$$

where Y is the vector of the observed milk yield, ***β*** is the vector of coefficients for fixed effects which included herd, breed group, age at calving in months, year of calving, season of calving and weeks in milk. j corresponds to environmental (THI) level, $f\left(\rho ,j\right)$ represents the population reaction norm function describing the relationship between the average animal performance and environment *j*, $f_{i}\left(\alpha ,j\right)$ corresponds to the individual cow reaction norm, describing the relationship between individual cow ***i*** and the environment *j*, ysm represents random effects of the YS of milking and e corresponds to the residual .X and Z correspond to design matrices for fixed and random ysm effects, respectively. Reaction norm functions were fitted using a Legendre polynomial of the second degree. The choice of Legendre polynomial of order 2 was based on a preliminary analysis that examined orders 1–3 to discover which one gave the best fit using the Akaike information criterium and likelihood ratio test.

Therefore, the population and individual reaction norm are given by Models (2) and (3) below:

(2) $$f_{p}=\rho_{0}+\rho_{1}x+\rho_{2}x^{2}+e$$

(3) $$f_{i}=\alpha_{0}+\alpha_{1}+\alpha_{2}x^{2}+e$$

Where $f_{p}$ and $f_{i}$ are the population and individual reaction norm models, $\rho_{0}$, $\rho_{1}$ and $\rho_{2}$ represent the intercept, linear coefficient and quadratic coefficient of the population reaction norm model. $\alpha_{0}$, $\alpha_{1}$ and $\alpha_{2}$ correspond to the intercept, linear regression coefficient and quadratic regression coefficient of the individual reaction norm model and x is the scaled THI value.

From the Models (2) and (3), the animal reaction norm is given by:

(4) $$f_{\text{anim}}=\left(\rho_{0}+\alpha_{0}\right)+\left(\rho_{1}+\alpha_{1}\right)x+\left(\rho_{2}+\alpha_{2}\right)x^{2}+e$$

The slope, and the absolute slope and the intercept of this animal reaction norm model were considered as an animal's resilience phenotypes for heat stress. The slope of the animal reaction norm was determined as the relative steepness of change in the response of each cow's milk yield to heat load at a given THI level. It was estimated as the derivative corresponding to a certain THI on the individual's response curve. Thus, the slope of the reaction norm is given by:

(5) $${\text{Slope}}_{\mathrm{rn}}=\left(\rho_{1}+\alpha_{1}\right)+2\left(\rho_{2}+\alpha_{2}\right)x$$

And the absolute slope of the reaction norm is given by:

(6) $${\text{Absolute}}_{\mathrm{rn}}=\left|\left(\rho_{1}+\alpha_{1}\right)+2(\rho_{2}+\alpha_{2}\right)x|$$

The reference THI was set to THI 80 to represent heat‐stress conditions (Oloo et al. 2024). Thus, x used to determine the slope of the reaction norm and its absolute value in this analysis was scaled THI 80. Because animal models assume that the trait analysed is normally distributed, the distribution of the absolute slope was normalised by applying a square root transformation before proceeding to subsequent genetic analyses.

For indicators based on deviations in milk yield (*LnVar1, LnVar2*, *r*_auto_ and *Skew*), the lactation curve for each cow based on test‐day milk yield was first fitted to predict the expected milk yield of a cow on each day in the absence of disturbances. A fourth‐order polynomial quantile regression model defined below was used to model the lactation curve for each cow using a quantile of 0.7. This method made the curves less sensitive to drops in milk yield and thus closer to the potential curves in the absence of disturbances (Koenker 2005).

(7) $${\text{yield}}_{t}=\beta_{0}+\beta_{1}t+\beta_{2}t^{2}+\beta_{3}t^{3}+\beta_{4}t^{4}+\varepsilon \;t$$

where ${\text{yield}}_{t}$ is the observed milk yield on *t*th days in milk (DIM *t*) and *ε* is the error term. The observed milk production and expected milk yield from the lactation curves were used to calculate actual and standardised deviation of *j*th animal on *i*th test‐day as shown below:

(8) $$\text{Actual}\;{\text{Deviation}}_{\mathrm{ij}}=\text{Observed}\;{\mathrm{MY}}_{\mathrm{ij}}-\text{Expected}\;{\mathrm{MY}}_{\mathrm{ij}}$$

(9) $${\text{Standardized Deviation}}_{\mathrm{ij}}=\frac{\text{Observed}\;{\mathrm{MY}}_{\mathrm{ij}}-\text{Expected}\;{\mathrm{MY}}_{\mathrm{ij}}}{\text{Expected}\;{\mathrm{MY}}_{\mathrm{ij}}}$$

The actual deviations of milk yield were used to define *LnVar1*, and standardised deviations were used to define *LnVar2*, *r*_auto_ and *Skew* resilience indicators, as previously described (Oloo, Mrode, et al. 2023).

Measurements of all resilience indicators of individual cows that deviated more than four SD from the mean were set to missing.

### 2.3 Statistical Analyses

#### 2.3.1 Resilience Indicators and Age at First Calving

Animal models with different fixed effects terms were fitted for each resilience indicator and AFC. Analysis of variance was used to determine significant factors of variation to be included in each model. For resilience indicators: *LnVar1, LnVar2*, *r*_auto_ and *Skew*, the fixed effect terms of the models included: breed, agroecology, age at calving, year of calving, season of calving, number of milk observations and class of days in milk of the first and last milk yield observation. In addition to these fixed effects, the average expected milk yield was fitted for *LnVar1*. The fixed effects fitted for *Slope*, *Absolute* and *Intercept* were breed, agroecology, age at calving, year of calving, season of calving and number of weekly milk records used to define the indicator. For AFC, the fixed effect terms were breed, agroecology, year, season of birth and year of calving.

The animal model below was fitted:

(10) y=Xβ+Za+e

where ***y*** is the vector of an animal observation for the analysed trait, ***β*** is the vector of coefficients for fixed effects, ***a*** is the additive genetic effects and ***e*** is the random residual effects. ***X*** and ***Z*** are the incidence matrices relating observation to fixed and random additive genetic effects, respectively. The vector of random additive genetic effects a and residual effects ***e*** were assumed to follow a normal distribution with a ~ *N* (*0*; ${A\sigma }_{a}^{2}$) and e ~ *N* (*0*; ${I\sigma }_{e}^{2}$), respectively, where ***I*** corresponds to the identity matrix and ***A*** corresponds to the numerator (A) relationship matrix, $\sigma_{a}^{2}$ is the additive genetic variance and $\sigma_{e}^{2}$ is the residual variance.

#### 2.3.2 Calving Interval

A repeatability animal model shown below was fitted for the calving interval:

(11) y=Xβ+Za+Wpe+e

where ***y*** is the vector of an animal observation for calving interval, ***β*** is the vector of coefficients for fixed effects consisting of agroecology, breed, year season of calving and parity nested within the age of the animal. **a**, pe and ***e*** are the additive genetic, permanent environmental and random residual effects, respectively. X,ZandW are the design matrices relating observation to fixed, random additive genetic and permanent environmental effects, respectively. It was assumed that the vector of random permanent environment effects pe follow a normal distribution with pe ~ *N* (*0*; ${I\sigma }_{\mathrm{pe}}^{2}$) where ***I*** correspond to the identity matrix and $\sigma_{\mathrm{pe}}^{2}$ is the permanent environment. The other assumptions were as in Model (10) above.

#### 2.3.3 Test‐Day Milk Yield

A fixed regression model (FRM) was implemented for daily milk yield data. The FRM in matrix form was as follows:

(12) $$y={\mathrm{X\beta}}_{1}+{\Phi \beta }_{2}+\mathrm{Za}+\mathrm{Wpe}+e$$

where ***y*** is the vector of an animal's daily milk yield observations, $\beta_{1}$ is the vector of coefficients for fixed effects consisting of herd, breed, year season of calving, year season of milking, parity, age effects nested within parity. Although five parities were represented in the data, parity effects were fitted as only four classes: parities 1, 2 and 3 separately, and parities 4 and 5 pooled into the fourth class. $\beta_{2}$ are vectors of parameters for the fixed lactation curves modelled by legendre polynomials of order 2; **a**, pe and ***e*** are the vectors of the random additive genetic effects, permanent environmental effects and residual effects, respectively. X,ZandW are the design matrices relating observation to fixed, random additive genetic and permanent environmental effects, respectively and Φ is the matrix of legendre polynomials of order 2 to model fixed lactation curves. The choice of legendre polynomial of order 2 was based on a preliminary analysis that examined several orders (1–5) to discover which gave the best fit using Akaike and Schwarz's Bayesian information criteria (Wolfinger 1993). The assumptions of this model were similar to those of Model (11) above.

#### 2.3.4 Bivariate Analyses

Genetic correlations between different resilience indicators, between resilience indicators and AFC, calving interval and test‐day milk yield were estimated using variances and covariances derived from the following bivariate mixed animal model:

(13) $$\left[\begin{array}{c} y_{1}\\ y_{2} \end{array}\right]=\left[\begin{array}{cc} X_{1} & 0\\ 0 & X_{2} \end{array}\right]\left[\begin{array}{c} b_{1}\\ b_{2} \end{array}\right]+\left[\begin{array}{cc} Z_{1} & 0\\ 0 & Z_{2} \end{array}\right]\left[\begin{array}{c} a_{1}\\ a_{2} \end{array}\right]+\left[\begin{array}{c} e_{1}\\ e_{2} \end{array}\right]$$

where $y_{i}$ is the vector with observations on trait *i*; $b_{i}$ is the vector of the fixed effects for trait *i*, identical to those included in the corresponding univariate analysis of the same trait; $a_{i}$ represents the additive genetic effects for trait *i* and $e_{i}$ is the residuals for trait *i*; $X_{i}$ and $Z_{i}$ are incidence matrices linking the records in $y_{i}$ to the fixed effects and additive genetic effects, respectively. The additive genetic effects for all traits were assumed to follow a normal distribution with a mean of 0, a genetic variance of $\sigma_{a_{i}}^{2}$ for trait *i* and a genetic covariance of $\sigma_{a_{1}a_{2}}$: $\left[\begin{array}{c} a_{1}\\ a_{2} \end{array}\right]$ ~ *N* $\left[\left(\begin{array}{c} 0\\ 0 \end{array}\right),A⊗\left[\begin{array}{cc} \sigma_{a_{1}}^{2} & \sigma_{a_{1}a_{2}}\\ \sigma_{a_{1}a_{2}} & \sigma_{a_{2}}^{2} \end{array}\right]\right]$. The residuals were assumed to be normally distributed with a mean of 0, a residual variance of $\sigma_{e_{i}}^{2}$ for trait *i* and a residual covariance between $\sigma_{e_{1}e_{2}}$: $\left[\begin{array}{c} e_{1}\\ e_{2} \end{array}\right]$ ~ *N* $\left[\left(\begin{array}{c} 0\\ 0 \end{array}\right),I⊗\left[\begin{array}{cc} \sigma_{e_{1}}^{2} & \sigma_{e_{1}e_{2}}\\ \sigma_{e_{1}e_{2}} & \sigma_{e_{2}}^{2} \end{array}\right]\right]$.

In addition, models for calving interval and test‐day milk yield had the permanent environmental effects fitted as $\left[\begin{array}{cc} W_{1} & 0\\ 0 & W_{2} \end{array}\right]\left[\begin{array}{c} {\mathrm{pe}}_{1}\\ {\mathrm{pe}}_{2} \end{array}\right]$ with ${\mathrm{pe}}_{i}$ as the vectors of the random permanent environmental effects and $W_{i}$ as the design matrices relating observations to permanent environmental effects. The environmental effects for all traits were assumed to follow a normal distribution with a mean of 0, an environmental variance of $\sigma_{{\mathrm{pe}}_{i}}^{2}$ for trait *i* and environmental covariance between $\sigma_{{\mathrm{pe}}_{1}{\mathrm{pe}}_{2}}$: $\left[\begin{array}{c} {\mathrm{pe}}_{1}\\ {\mathrm{pe}}_{2} \end{array}\right]$ ~ *N* $\left[\left(\begin{array}{c} 0\\ 0 \end{array}\right),I⊗\left[\begin{array}{cc} \sigma_{{\mathrm{pe}}_{1}}^{2} & \sigma_{{\mathrm{pe}}_{1}{\mathrm{pe}}_{2}}\\ \sigma_{{\mathrm{pe}}_{1}{\mathrm{pe}}_{2}} & \sigma_{{\mathrm{pe}}_{2}}^{2} \end{array}\right]\right]$. The likelihood ratio test was used to determine whether genetic correlations among resilience indicators were significantly different from zero, by comparing the modelled equation to a bivariate model with additive genetic covariance fixed at zero.

All the animal models were fitted in R (version 4.4.1; R Core Team 2024) using the ASReml‐R 4.2 package (Butler et al. 2023).

## 3 Results

### 3.1 General Data Summary

Table 1 represents the descriptive statistics of all the resilience indicators analysed.

**TABLE 1:** Descriptive statistics of resilience phenotypes based on first parity milk production of dairy cows.

Resilience indicator	Count	Mean	SD	Minimum	Maximum
*Slope*	1543	−0.039	0.517	−2.045	1.976
*Absolute*	1543	0.569	0.264	0.017	1.430
*Intercept*	1544	0.066	2.187	−8.775	9.824
*LnVar1*	2368	0.524	0.650	−1.809	2.477
*LnVar2*	2360	−4.020	0.737	−6.530	−1.216
*r*_auto_	2372	0.305	0.248	−0.405	0.918
*Skew*	2362	−0.336	1.045	−5.347	5.262

**FIGURE 1:**
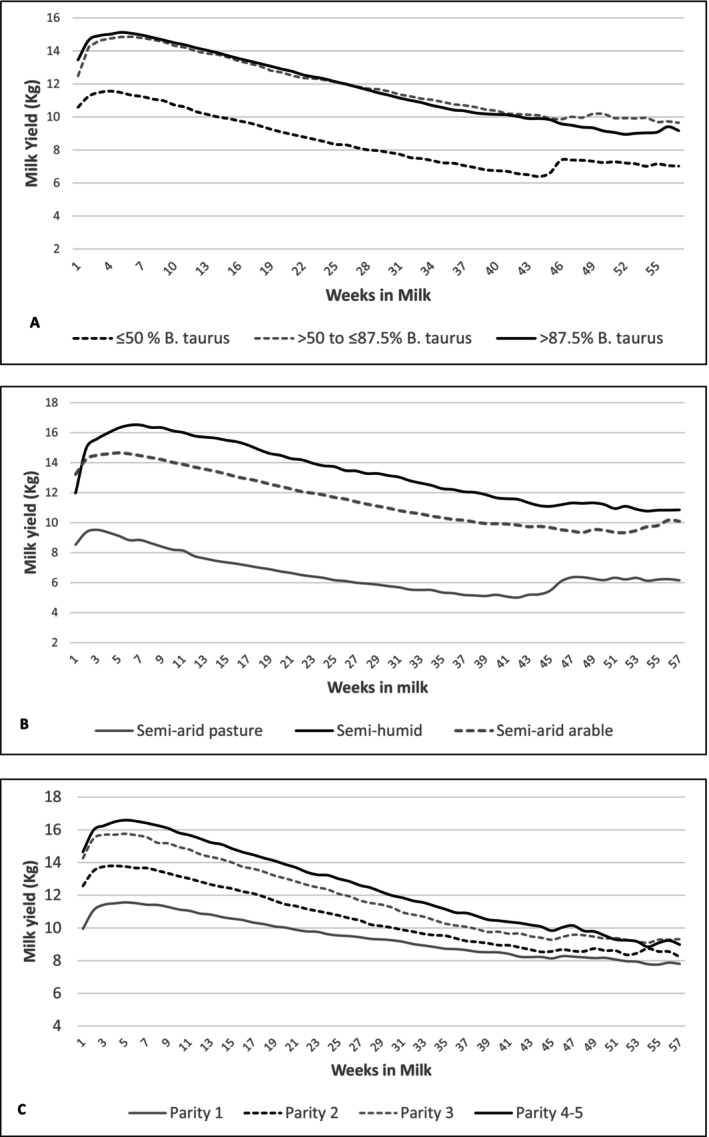
Average milk yield by weeks in milk for (A) different breeds, (B) different performing environments and (C) different parities of cows under study.

Table 2 represents summary statistics of AFC, calving interval and test‐day milk yield of the cows under study, and Figure 1 shows simple plots of daily milk yield by weeks in milk for different breeds, environments and four parity classes. Cows with ≤ 50% taurine genes recorded the highest average AFC, while no marked differences in AFC were observed among cows with > 50%–≤ 87.5% and > 87.5% taurine genes. Animals performing in SH agroecological zone had the lowest average AFC. The animals with ≤ 50% *Bos taurus* had the lowest calving interval. Animals performing in semi‐arid conditions had significantly shorter calving intervals than those in the SH environment. Calving interval shortened as the number of parity increased. The average daily milk yield for cows with > 50%–≤ 87.5% and > 87.5% taurine genes was similar but significantly higher than that of cows with ≤ 50% *B. taurus* genes. Animals in the SH environment produced more milk than those performing in two semi‐arid environments. As expected, mean milk yield showed an increasing trend as the number of parity increased.

**TABLE 2:** Summary statistics showing number of records (*N*) and least‐square means ± standard error (Mean) of age at first calving, calving interval and milk yield of dairy cows under study.

Factor and level	Age at first calving		Calving interval		Test‐day milk yield	
	*N*	Mean	*N*	Mean	*N*	Mean
Breed group						
≤ 50% *B. taurus*	1118	29.4 ± 0.14^a^	1581	455 ± 2.96^a^	72,955	9.9 ± 0.11^a^
> 50%–≤ 87.5% *B. taurus*	817	29 ± 0.14^b^	1330	483 ± 3.16^b^	60,780	12.6 ± 0.12^b^
> 87.5% *B. taurus*	1570	28.7 ± 0.11^bc^	3280	486 ± 2.88^bc^	143,046	12.5 ± 0.10^bc^
Herd						
Semi‐arid pasture zone	1006	29.9 ± 0.15^a^	1159	468 ± 3.18^a^	41,757	7.7 ± 0.14^a^
Semi‐humid zone	558	27.6 ± 0.16^b^	576	500 ± 4.46^b^	32,188	15.4 ± 0.15^b^
Semi‐arid arable zone	1941	29.5 ± 0.12a^c^	4456	457 ± 2.41^c^	202,836	11.9 ± 0.07^c^
Parity						
1					94,386	11.1 ± 0.10^a^
2			2275	590 ± 4.99^a^	60,375	11.2 ± 0.07^a^
3			1697	448 ± 2.26^b^	55,498	11.9 ± 0.07^b^
4 and 5			2219	386 ± 3.39^c^	66,522	12.5 ± 0.08^c^

*Note:* Least square means sharing no superscript letter are significantly different.

### 3.2 Genetic Parameters of Fertility Traits and Milk Yield

The variance components of AFC, calving interval and test‐day milk yield are presented in Table 3. The two fertility traits had significant heritability estimates, revealing the possibility of improving these traits through breeding. Calving interval had the lowest heritability estimate, and its permanent environmental variance was not significant. Test‐day milk yield had a moderate heritability estimate of 0.35 and a repeatability of 0.47. The variance due to the permanent environment was significant. There was a significant high negative genetic correlation of −0.876 ± 0.077 between AFC and CI, indicating that animals that attained sexual maturity earlier faced challenges in maintaining shorter inter‐calving durations. A significant moderate negative genetic correlation of −0.526 ± 0.059 observed between test‐day milk yield and AFC reveals an earlier onset of puberty in higher‐producing cows. Test‐day milk yield had a moderate positive genetic correlation with calving interval (0.617 ± 0.077), which shows that the higher the production potential of an animal, the longer the interval between two consecutive calving events. Thus, higher‐producing animals had lower AFC and longer calving intervals.

**TABLE 3:** Variance parameters (±SE) of age at first calving (AFC), calving interval (CI) and test‐day milk yield (MY).

Trait	Additive variance	Environment variance	Residual variance	Phenotypic variance	Heritability	Repeatability
AFC	1.5 ± 0.31		7.4 ± 0.3	8.9 ± 0.23	0.17 ± 0.033*	
CI	363.2 ± 73.17	0.001 ± 0	5432.1 ± 112.1	5795.3 ± 107.6	0.06 ± 0.012*	0.06 ± 0.012*
MY	5.13 ± 0.37	1.76 ± 0.21	7.78 ± 0.02	14.67 ± 0.24	0.35 ± 0.021*	0.47 ± 0.009*

*Note:* Estimates that differ significantly from zero at *p* < 0.05 have been marked by an asterisk.

### 3.3 Genetic Parameters of Resilience Indicators

**FIGURE 2:**
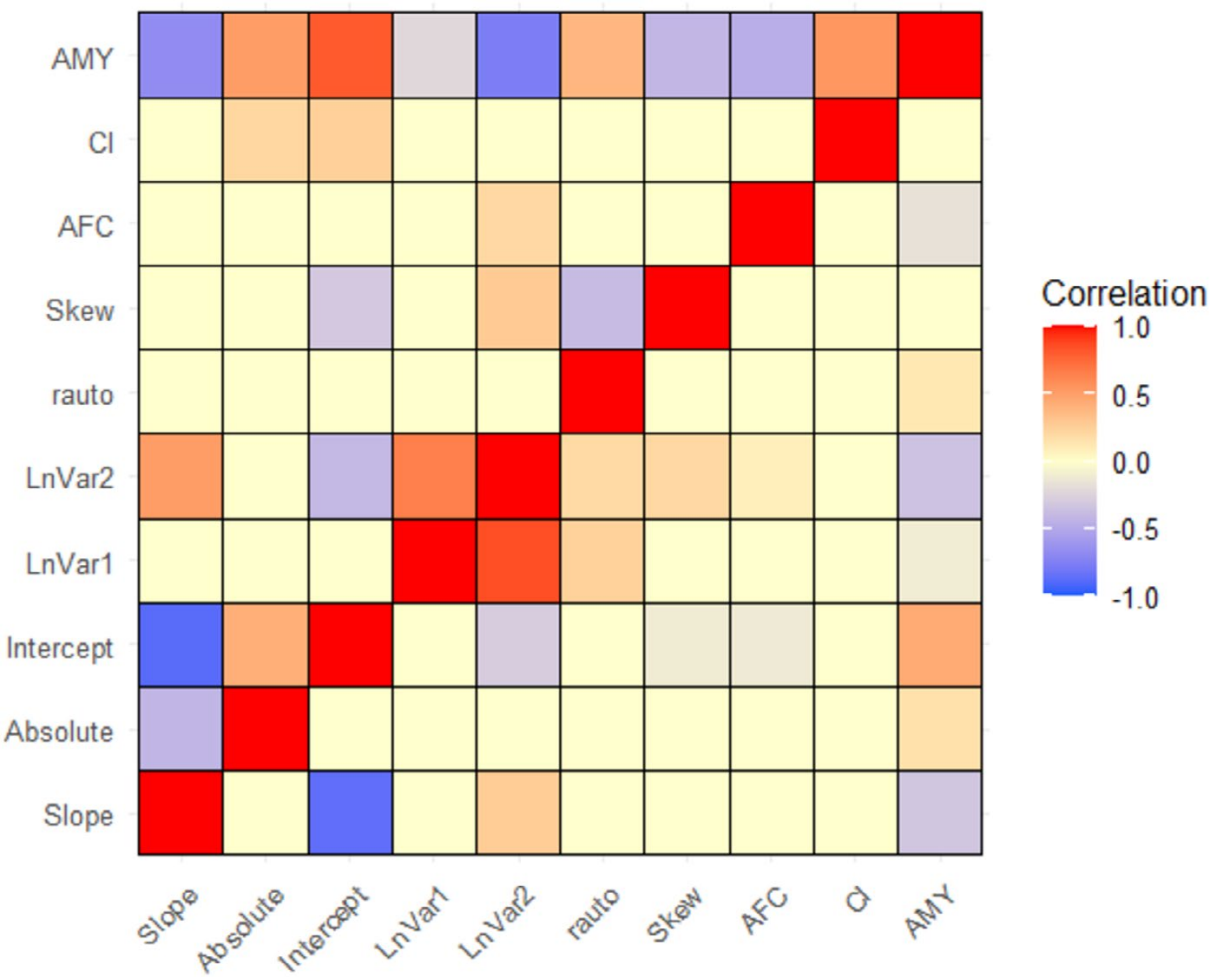
A correlation heatmap showing relationships among all resilience indicators, fertility traits and milk yield (AMY). Above diagonal shows genetic correlations and below diagonal shows phenotypic correlations. The colours represent the strength of correlations.

Variance components of resilience indicators are presented in Table 4. All the traits except *Skew* had heritability estimates that significantly differed from zero. *Slope* had the highest, while *Skew* had the lowest heritability estimates. Genetic correlations among the resilience indicators have been shown in Table 5 and Figure 2.

**TABLE 4:** Variance parameters (±SE) of resilience phenotypes.

Trait	Additive variance	Residual variance	Phenotypic variance	Heritability
*Slope*	0.089 ± 0.016	0.171 ± 0.013	0.261 ± 0.01	0.34 ± 0.054*
*Absolute*	0.01 ± 0.003	0.055 ± 0.003	0.066 ± 0.002	0.16 ± 0.05*
*Intercept*	1.289 ± 0.265	3.274 ± 0.235	4.563 ± 0.176	0.28 ± 0.054*
*LnVar1*	0.033 ± 0.009	0.211 ± 0.01	0.244 ± 0.007	0.14 ± 0.037*
*LnVar2*	0.089 ± 0.016	0.171 ± 0.013	0.29 ± 0.009	0.24 ± 0.043*
*r*_auto_	0.002 ± 0.001	0.029 ± 0.001	0.031 ± 0.001	0.08 ± 0.031*
*Skew*	0.049 ± 0.03	0.923 ± 0.038	0.972 ± 0.029	0.05 ± 0.03

*Note:* Heritability estimates that were significantly different from zero at *p* < 0.05 have been marked by an asterisk.

**TABLE 5:** Genetic (above diagonal) and phenotypic (below diagonal) correlations (±SE) among resilience indicators.

Trait	*Slope*	*Absolute*	*Intercept*	*LnVar1*	*LnVar2*	*r*_auto_	*Skew*
*Slope*		−0.57 ± 0.149*	−0.91 ± 0.026*	0.04 ± 0.173	0.51 ± 0.12*	0.32 ± 0.203	0.38 ± 0.271
*Absolute*	−0.13 ± 0.026		0.59 ± 0.165*	0.09 ± 0.24	−0.26 ± 0.18	−0.14 ± 0.276	−0.34 ± 0.346
*Intercept*	−0.87 ± 0.007	0.11 ± 0.026		0.06 ± 0.187	−0.41 ± 0.135*	−0.33 ± 0.212	−0.61 ± 0.304*
*LnVar1*	0.04 ± 0.029	0.03 ± 0.028	−0.12 ± 0.3		0.66 ± 0.075*	0.1 ± 0.236	0.12 ± 0.298
*LnVar2*	0.26 ± 0.026*	−0.06 ± 0.027	−0.31 ± 0.025	0.85 ± 0.007*		−0.16 ± 0.221	0.51 ± 0.231*
*r*_auto_	−0.01 ± 0.027	0.04 ± 0.027	0.01 ± 0.027	0.24 ± 0.02*	0.2 ± 0.021*		−0.613 ± 0.224*
*Skew*	0.1 ± 0.027	−0.06 ± 0.027	−0.14 ± 0.027*	0.00 ± 0.214	0.21 ± 0.021*	−0.01 ± 0.021	

*Note:* Significant correlations have been marked with an asterisk.

A negative correlation between *Slope* and *Absolute* showed that, under high heat load conditions, animals with a generally more favourable response in milk yield have a more stable milk production profile. *Slope* and *Absolute* had a high negative and a low positive correlation with *Intercept*, respectively. This showed that higher milk‐producing cows have more negative and less stable milk production when under thermal stress. *LnVar2* had a significant positive correlation with *Skew*, insinuating that animals deemed more resilient using *LnVar2* were less resilient when skew is used as a resilience indicator. *Skew* exhibited a negative correlation with *r*_auto_, suggesting that a resilient animal using *Skew* is likely to be resilient using *r*_auto_.

*Slope* had a significant positive correlation with both *LnVar2* and *r*_auto_, meaning that animals whose milk production responded more favorably in heat stress conditions had a higher general resilience. According to *LnVar2*, animals exhibiting a higher degree of resilience to day‐to‐day disturbance were generally producing more milk, as indicated by the negative correlation between the indicator and the *Intercept*. However, a negative correlation observed between the *Intercept* and *Skew* shows that animals with higher general resilience based on *Skew* produce lower milk yield. *Absolute* did not have a significant correlation with all three indicators of resilience to day‐to‐day disturbances.

### 3.4 Relationship Between Resilience and Performance Traits

Table 6 presents genetic and phenotypic correlations of resilience indicators and fertility and milk production traits. All heat tolerance indicators had non‐significant genetic correlations with AFC. Although the correlations were not significant, their direction showed that cows that are tolerant to high heat load may have a higher AFC and vice versa. For indicators of general resilience, only *LnVar* had a significant positive correlation with AFC, showing that animals exhibiting resilience to day‐to‐day disturbances had a low AFC.

**TABLE 6:** Genetic and phenotypic correlations (±SE) of resilience indicators with age at first calving, calving interval and test‐day milk yield.

Resilience Indicator	Age at first calving		Calving interval		Test‐day milk yield	
	*r*A	*r*P	*r*_A_	*r*P	*r*A	*r*P
*Slope*	0.22 ± 0.144	0.077 ± 0.033	−0.176 ± 0.147	0.061 ± 0.032	−0.713 ± 0.039*	−0.35 ± 0.024*
*Absolute*	−0.315 ± 0.199	−0.059 ± 0.033	0.415 ± 0.2*	0.001 ± 0.034	0.606 ± 0.096*	0.189 ± 0.027*
*Intercept*	−0.174 ± 0.155	−0.151 ± 0.033	0.402 ± 0.152*	−0.007 ± 0.033	0.838 ± 0.029*	0.467 ± 0.02*
*LnVar1*	−0.219 ± 0.182	−0.001 ± 0.027	0.258 ± 0.168	0.024 ± 0.026	−0.249 ± 0.025*	−0.131 ± 0.025*
*LnVar2*	0.347 ± 0.139*	0.111 ± 0.025*	−0.045 ± 0.14	0.058 ± 0.026	−0.805 ± 0.041*	−0.368 ± 0.02*
*r*_auto_	0.14 ± 0.218	−0.029 ± 0.025	0.163 ± 0.212	0.069 ± 0.026	0.507 ± 0.124*	0.147 ± 0.022*
*Skew*	0.093 ± 0.28	0.048 ± 0.025	−0.411 ± 0.267	−0.008 ± 0.026	−0.558 ± 0.142*	0.022 ± 0.023*

*Note:* Significant correlations at *p* < 0.05 have been marked with an asterisk.

Calving interval was moderately positively correlated with *Intercept*. This implies that the animals with higher mean milk production in response to high heat load had longer calving intervals and vice versa. The significant positive correlation between calving interval and *Absolute* suggests that animals with a more stable milk production under heat stress conditions were inclined to have shorter calving intervals. All other resilience indicators were not significantly correlated with calving intervals.

All the indicators of resilience had significant genetic and phenotypic correlations with test‐day milk yield. A high negative genetic correlation observed between *Slope* and *MY* showed that cows having a more favourable response of milk yield to heat stress produced lower test‐day milk yield. *Absolute* had a positive correlation with milk yield, revealing that cows with more stable milk production in the face of heat stress were inclined to produce lower milk yield. *Intercept* was also positively correlated with test‐day milk yield, as expected. *LnVar1* had a significantly low negative correlation, while *LnVar2* had a high negative correlation with test‐day milk yield. This shows that based on the variance of deviations indicators, animals with higher resilience to general disturbances exhibit higher test‐day milk production. The *r*_auto_ had a moderate positive correlation and *Skew* had a moderate negative correlation with test‐day milk yield. This shows that, according to *r*_auto_ and *Skew*, more resilient cows produce lower milk yield.

## 4 Discussion

Increasing dairy productivity requires simultaneous and appropriately weighted improvements of milk production, adaptability to the production environment, improved health, fertility, efficiency and growth performance. In middle‐ and low‐income regions, the lack of sufficient data has impeded the inclusion of all these traits in the breeding objectives (Oloo, Ojango, et al. 2023). At the moment, most genetic evaluations in these regions consider only milk production and a few other traits in their selection index (Mrode et al. 2021). However, with the ongoing climate change and its adverse effects on the environment, it is imperative to factor in resilience and robustness in the breeding objectives to ensure the long‐term sustainability and profitability of the dairy industry. A better understanding of the relationship between resilience and the key traits that are already being bred for opens an opportunity for including resilience in the selection indices. This study examined the relationship of different resilience indicators with milk production, AFC and calving interval in dairy cows performing in Kenya.

The proportion of taurine genes in the genetic makeup of dairy cows influenced both fertility and milk production performance. AFC decreased with an increase in the percentage of taurine genes in the genotype of an animal. This observation could be attributed to differential growth rates in the animals with different proportions of taurine genes. Cattle with higher taurine genetics have been reported to grow faster (Lukuyu et al. 2016; Duplessis et al. 2015; Krpálková et al. 2014); hence, they attain sexual maturity earlier than animals whose genetic composition comprises fewer taurine genes. A longer calving interval was observed in cows with a high proportion of taurine genes. The deterioration in fertility in high‐grade crossbred cows could be associated with lowered adaptation following a decrease in heterozygosity and genes of indicine origin in such crosses (Oloo et al. 2022; Oloo, Ojango, et al. 2023; Bahmani et al. 2011). Animals with a higher proportion of taurine genes had a higher average test‐day milk production as expected. In all the fertility and production traits analysed, there was no significant difference in performance between cows with > 50%–87.5% and > 87.5% taurine genes. Thus, considering adaptability issues and related management costs of high‐grade cattle (Oloo, Ojango, et al. 2023; Mwai et al. 2015), it might be profitable to breed for cows with > 50%–87.5% taurine genes in this region.

Animals raised in semi‐arid environments had a later AFC, shorter calving intervals and lower milk production than those in the SH environment. These differences in AFC and milk production can be partly attributed to the differences in climatic conditions across these environments. Semi‐arid environments are characterised by low rainfall and frequent droughts, which cause shortages in feed and water quality and quantity available to the animals, hence making them prone to new diseases, low milk production and slow growth rates (Rojas‐Downing et al. 2017; Nardone et al. 2010; Thornton 2010). Longer calving intervals observed in cows performing in the SH region were partly because of the deliberate breeding decisions taken by the farmer that ensured cows calved when the demand for milk was high and the market was readily available (Oloo et al. 2022). Besides, since the animals performing in the SH zone produced more milk yield than those in semi‐arid regions, longer calving intervals could be associated with higher lactational stresses, including the associated higher mobilisation of body reserves, hence more loss of body condition in their early post‐partum periods to meet and maintain milk production needs (Roche et al. 2009; Leroy et al. 2008). This leads to a prolonged duration before the first post‐partum estrus is displayed (Butler 2003; Broster and Broster 1998) leading to longer days open.

The substantial heritability estimates of two fertility traits and test‐day milk yield imply a promising opportunity for selection and improvement of these traits within the dairy cattle population in this region. AFC had a heritability estimate of 0.17, which was similar to estimates found in the literature (Shi et al. 2021; Berry et al. 2014). Calving interval had a low but significant heritability estimate of 0.06, and its permanent environment variance was not significant. This suggests that the trait is majorly influenced by extrinsic factors, such as management practices adopted and the prevailing climatic conditions. The heritability and repeatability estimates of milk yield (0.35 and 0.47, respectively) were within the previously reported ranges for cows (Ojango et al. 2019; Pritchard et al. 2013).

The antagonistic relationship between AFC and calving interval inferred different underlying causes for each trait, despite both traits being considered fertility traits. The AFC is influenced more by genetic factors that influence growth since the onset of puberty is determined by body weight (Van Amburgh et al. 2019; Coffey et al. 2006). Therefore, animals with faster growth rates develop faster and reach puberty earlier, resulting in a lower AFC. A negative correlation existed between milk yield and AFC. High milk‐producing animals tend to grow faster and have large body sizes, which help them attain sexual maturity at an earlier age, resulting in a lower AFC. A positive genetic correlation between milk yield and calving interval suggests that higher‐yielding animals had longer inter‐calving intervals. This can be explained by the mobilisation of body reserves for milk production and negative energy balance in high‐yielding cows, which affects the health of the animals. As explained by Dobson et al. (2008), establishing a pregnancy is risky for a cow in poor health, so through a survival instinct, cows will not show obvious signs of estrus to avoid conception, resulting in prolonged calving intervals.

Indicators of resilience to macro‐environmental disturbances were based on the response of milk yield to high thermal conditions of THI 80 and included *Slope*, *Absolute* and *Intercept*. High *Slope* and low *Absolute* were expected to indicate high resilience, which means low vulnerability to heat stress and high stability of milk production under heat stress conditions (Sánchez‐Molano et al. 2019). All these indicators had a significant heritability estimate denoting a possibility of using them to breed for heat tolerance in dairy cows. Their heritability estimates ranged from 0.16 to 0.34 and were within the range previously reported (Oloo et al. 2024; Tsartsianidou et al. 2021; Sánchez‐Molano et al. 2019). A negative correlation was observed between *Slope* and *Absolute* at high heat load conditions (THI 80). This suggests that resilient cows have a more stable milk production under heat stress conditions and that their milk production responds more favourably to high heat load conditions. The intercept of the reaction norm model had a positive and a negative correlation with *Slope* and *Absolute*, respectively. As *Intercept* represents the mean milk production, this finding shows that higher milk‐producing cows are more vulnerable to heat stress and portray a less stable milk production profile when under thermal stress.

Indicators of resilience to microenvironmental disturbances were based on standardised deviations in milk yield of animals and included log‐transformed variance (*LnVar1* and *LnVar2*), lag‐1 autocorrelation (*r*_auto_) and skewness (*Skew*) in deviations of milk yield. Low *LnVar*, low *r*_auto_ and high *Skew* were expected to show better resilience to general disturbances (Poppe et al. 2020; Berghof et al. 2019). Only *Skew* did not have a significant heritability estimate. The heritability estimates of these indicators ranged from 0.5 to 0.24 and were within the range previously reported in dairy cows (Keßler et al. 2024; Oloo et al. 2023a; Poppe et al. 2020). *LnVar1* and *LnVar2* were moderately positively correlated, insinuating a substantial level of similarity between the two variances of deviations indicators. An antagonistic relationship was reported between the variance of actual deviations and the variance of standardised variation when the milk‐producing potential of the cows is not corrected for in the model (Oloo, Mrode, et al. 2023). These findings suggest that, similar to the standardisation of deviations in milk yield, correcting for the milk production potential of the animals reduces the chances of inaccurately concluding that lower‐producing animals are more resilient.

*LnVar2* was moderately positively correlated with *Skew*, which shows that an animal considered resilient using *LnVar2* may not be resilient when *Skew* is used as an indicator of resilience. This could mean that the two indicators are genetically different and capture different aspects of resilience (Oloo, Mrode, et al. 2023; Poppe et al. 2020). Indeed, the variance of deviations is believed to capture both the severity and duration, while *Skew* captures only the severity of environmental disturbance an individual experiences (Berghof et al. 2019). A moderate negative correlation between *Skew* and *r*_auto_ shows that cows with a faster rate of recovery from a disturbance are likely to have been less severely affected by the disturbance.

A general lack of significant correlations between indicators of resilience to heat stress and indicators of resilience to general disturbances could be partly explained by the underpowered design of this study and partly by differential aspects of resilience captured by these indicators. Different genes are involved in handling different disturbances. Heat stress, being a specific disturbance, its tolerance in the cells of cattle is mediated by a family of heat shock proteins (Kim et al. 2017). General disturbance, on the other hand, comprises all forms of unknown day‐to‐day disturbances that an animal experiences throughout the lactation (Berghof et al. 2019). Thus, a wider range of genes may be involved in resilience to general disturbances. However, given the limited number of observations in this study, further verification could be provided through follow‐up studies, with adequate observations.

The findings of our study revealed a positive correlation between resilience and fertility in dairy cows. *LnVar2* had a positive correlation with AFC, showing that when using this indicator to measure resilience, animals with high resilience tend to have a lower AFC and vice versa. Although not significant, *LnVar1* showed that resilient animals had shorter calving intervals. *Intercept* and *Absolute* were significantly correlated with calving interval. They show that animals with lower calving intervals had lower mean milk yield and stable milk production during high thermal conditions. Since heat stress negatively affects follicular development, estrus detection and conception rates in cows, leading to deteriorated fertility (Shi et al. 2021), animals with better heat tolerance are expected to have better fertility. Besides, resilient animals have been found to have generally better health (Poppe et al. 2020; Elgersma et al. 2018), thus avoiding fertility issues stemming from poor health. For instance, a disease early in lactation may impair the cow's ability to show estrus and to conceive after insemination. A disease like mastitis can affect the resumption of ovarian activity in post‐partum cows (Huszenicza et al. 2005).

Test‐day milk yield exhibited a significant correlation with all resilience indicators. It had a negative correlation with *Slope* and a positive correlation with *Absolute* and *Intercept*. This shows that high‐yielding animals have a less stable milk production during heat stress conditions and their milk production responds more negatively to heat stress (Oloo et al. 2024). *Skew* and *r*_*auto*_ had a moderate negative and positive correlation with milk yield, respectively. This shows that, according to these two indicators, more resilient animals produce lower milk yield. *LnVar1* and *LnVar2*, on the other hand, had a negative correlation with milk yield, which implied that animals with higher resilience to general disturbances have higher milk yield and vice versa. Similar observations were made by Oloo, Mrode, et al. (2023) and Keßler et al. (2024). The positive correlation between *LnVar1* and milk yield implies that correcting for average expected milk yield in the model prevents the variance of actual deviations from categorising inaccurately low‐producing animals as being resilient. Collectively, all indicators except variance of deviation indicators revealed an antagonistic relationship between resilience and milk production. This could be attributed to differential resource allocation in different cows. High‐yielding cows may have fewer resources available to respond to disturbances compared to low‐producing cows because of the high resource demand for their milk production (Rauw 2008). Resilient animals are more likely to divert resources away from milk production in response to disturbances and hence produce lower milk yield than non‐resilient animals. The findings on LnVar1 and LnVar2 are also biologically plausible; a lack of resilience, particularly in challenging environments, can cause an animal to produce well below its optimal level. In such scenarios, highly resilient cows are likely to outperform less resilient ones in terms of production. These contradictory findings call for further studies on resilience and resilience indicators as the indicators could be capturing different aspects of resilience.

## 5 Conclusions

Results from this study highlight the genetic relationship among resilience, fertility and production traits in dairy cattle. Cows with a higher proportion of taurine genes had a lower AFC, longer CI and higher milk yield. Cows performing in semi‐arid environments had a later AFC, shorter calving intervals and lower milk production. AFC was moderately negatively correlated with both calving interval and test‐day milk yield, which suggested that animals with early onset of puberty were likely to produce more milk yield and register longer CI in the long run. All indicators of resilience, except skewness of deviation, had significant heritability estimates. The poor correlation between indicators of heat tolerance and indicators of general resilience in this study insinuates that these indicators are genetically different and that different genes are involved in surviving these disturbances. This study found a positive association between resilience and fertility, suggesting that resilient animals have better fertility and vice versa. All indicators of resilience, except *LnVar1* and *LnVar2*, revealed an antagonistic relationship between resilience and milk production. This implies that resilient animals are likely to prioritise their survival amidst disturbance more than milk production. This study presents an opportunity for the inclusion of resilience indicators in the selection indices of dairy cattle, especially in tropical regions.

## Author Contributions

**R. D. Oloo:** conceptualisation, investigation, data curation, methodology, software, formal analysis, visualisation, writing – original draft, writing – review and editing. **R. Mrode:** conceptualisation, data curation, methodology, writing – review and editing, supervision. **C. C. Ekine‐Dzivenu:** data curation, methodology, writing – review and editing. **J. Bennewitz:** conceptualisation, methodology, writing – review and editing, supervision. **J. M. K. Ojango:** data curation, methodology, writing – review and editing. **G. Gebreyohanes:** data curation, writing – review and editing. **A. M. Okeyo:** conceptualisation, data curation, funding acquisition, writing – review and editing, supervision. **M. G. G. Chagunda:** conceptualisation, methodology, funding acquisition, writing – review and editing, supervision.

## Disclosure

Declaration of Generative AI and AI‐Assisted Technologies in the Writing Process: The authors did not use any artificial intelligence–assisted technologies in the writing process.

## Ethics Statement

The authors have nothing to report.

## Conflicts of Interest

The authors declare no conflicts of interest.

## Data Availability

The data/models were not deposited in an official repository. Data are available upon request to the corresponding author.
